# Supplementary material for: Targeting IKKβ Activity to Limit Sterile Inflammation in Acetaminophen-Induced Hepatotoxicity in Mice
Source: Pharmaceutics. 2023 Feb 20;15(2):710. doi: 10.3390/pharmaceutics15020710 (PMC9959252; doi:10.3390/pharmaceutics15020710)
Supplement: Supplementary file 1 [file pharmaceutics-15-00710-s001.zip › pharmaceutics-2180749-supplementary.pdf]

Supplementary materials

Targeting IKKβ Activity to Limit Sterile Inflammation in Acetaminophen-Induced Hepatotoxicity in Mouse.

Song-Hee Kim [1,†], Da-Eun Jung [1,†], Jin Yong Song [1], Jihye Jung [1], Jae-Kyung Jung [1], Heesoon Lee [1], Eunmiri Roh [2], Jin Tae Hong [1], Sang-Bae Han [1] and Youngsoo Kim [1,*]

[1]College of Pharmacy, Chungbuk National University, Cheongju 28160, Republic of Korea

[2]Department of Cosmetic Science, Kwangju Women's University, Gwangju 62396, Republic of Korea

*Correspondence: youngsoo@chungbuk.ac.kr

[†]These authors contributed equally to this work.

Contents

Table S1

Figures S1 to S5

Table S1. Nucleotide sequence of RT-PCR primer.

Target	Nucleotide sequence		Amplicon
CCL2	Forward	5'-CCAGCTCTCTCTTCCTCCAC-3'	494 bp
	Reverse	5'-AAGGCATCACAGTCCGAGTC-3'	
CXCL1	Forward	5'-GCTGGGATTCACCTCAAGAA-3'	469 bp
	Reverse	5'-TGATGGGCAGCAGGTCTCAT-3'	
CXCL2	Forward	5'-AAAGTTTGCCTTGACCCTGA-3'	498 bp
	Reverse	5'-CACCCCTTATCCCCAGTCTC-3'	
CYP1A2	Forward	5'-AGAGCGGTTTCTTACCAATA-3'	526 bp
	Reverse	5'-GCCAGAGTAGGCAAATCT-3'	
CYP2E1	Forward	5'-GATGAATATGCCCTACATG-3'	468 bp
	Reverse	5'-TGATGGGCAGCAGGTCTCAT-3'	
GSTP1	Forward	5'-ATGCCACCATACACCATTGTC-3'	161 bp
	Reverse	5'-GGGAGCTGCCCATACAGAC-3'	
GSTT1	Forward	5'-AGGCTCGTGCTCGTGTAGA-3'	96 bp
	Reverse	5'-CAGGGAACATCACCTTATGCC-3'	
IL-1β	Forward	5'-CCTGTCCTGTGTAATGAAAGACGGC-3'	526 bp
	Reverse	5'-GTGCTGCCTAATGTCCCCTTGAATC-3'	
IL-6	Forward	5'-CCGGAGAGGAGACTTCACAG-3'	134 bp
	Reverse	5'-CAGAATTGCCATTGCACAAC-3'	
TNF-α	Forward	5'-ATGAGCACAGAAAGCATGATCCGC-3'	644 bp
	Reverse	5'-CCAAAGTAGACCTGCCCGGACTC-3'	
β-Actin	Forward	5'-CACCACACCTTCTACAATGAGCTGC-3'	745 bp
	Reverse	5'-GCTCAGGAGGAGCAATGATCTTGAT-3'	

Abbreviation: bp, base pairs.

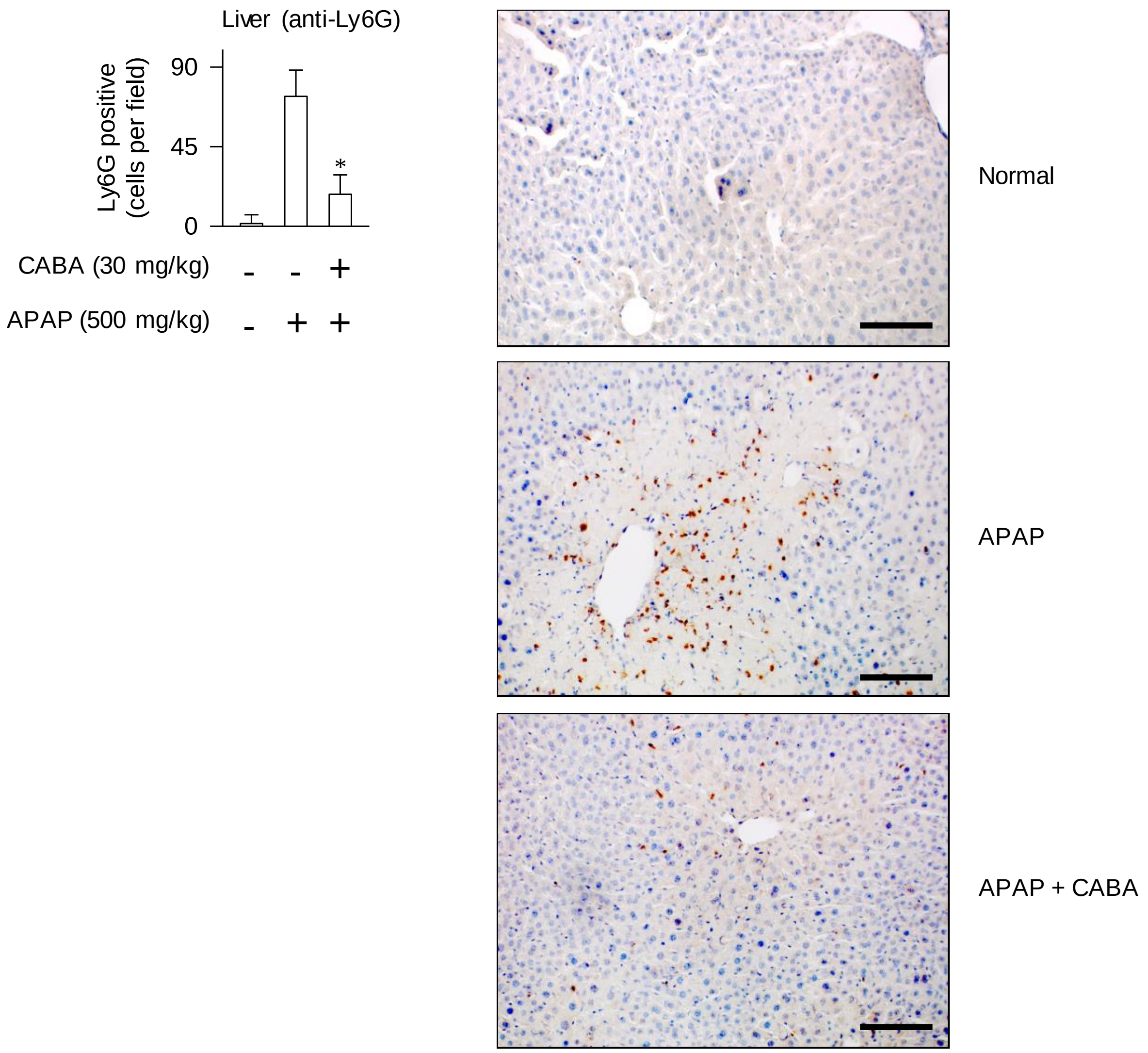

Figure S1. C57BL/6 mice were treated with CABA at 1 h after APAP overdose. Liver lobules were biopsied at the time lapse of 15 h. Liver tissues were sectioned in a thickness of 5 μm, reacted with anti-Ly6G antibody, and examined under microscope. Black scale bars are 100 μm. *$P < 0.05$ vs. APAP alone.

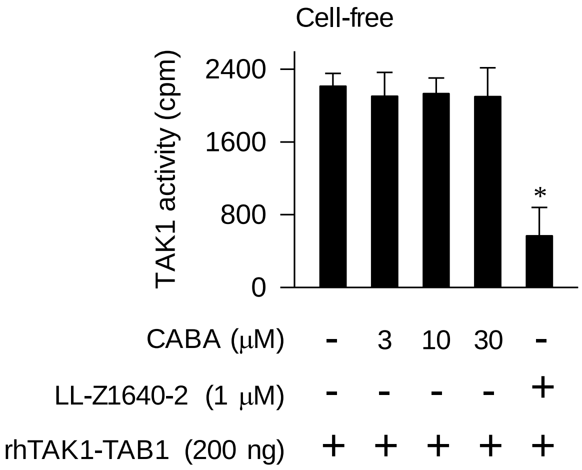

Figure S2. Catalytically active rhTAK1-TAB1 was treated with CABA or LL-Z1640-2 for 10 min in cell-free reactions. *In vitro* kinase activity was determined by the corporation of [^{32}P] onto MBP as exogenous substrate from [γ-^{32}P]-labeled ATP, and represented as count per min (cpm). LL-Z1640-2 was employed as positive control agent inhibiting TAK1 activity. *$P < 0.05$ vs. rhTAK1-TAB1 alone.

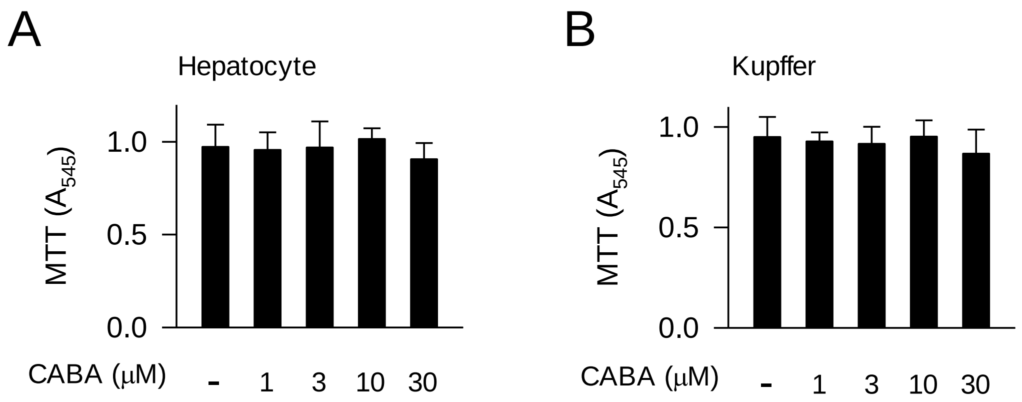

Figure S3. Primary hepatocytes (**A**) or Kupffer cells (**B**) were incubated with CABA for 24 h, and reacted with MTT for another 1 h. Formazan crystals were dissolved in 50% dimethyl sulfoxide and measured the absorbance values at wavelength 545 nm (A_{545}).

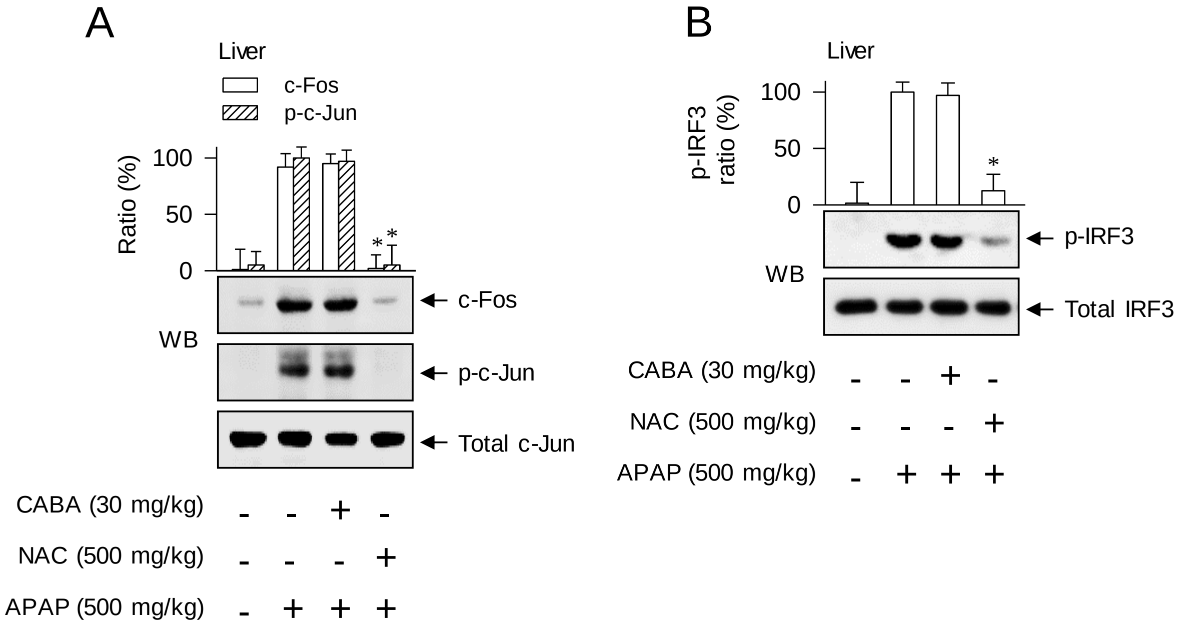

Figure S4. C57BL6 mice were treated with CABA at 1 h after APAP overdose. Liver lobules were biopsied at the time lapse of 15 h. Protein extracts were subjected to Western blot (WB) analysis with anti-Fos, anti-p-c-Jun or anti-c-Jun antibody (**A**) and anti-p-IRF3 or anti-IRF3 (**B**). *$P < 0.05$ vs. APAP alone.

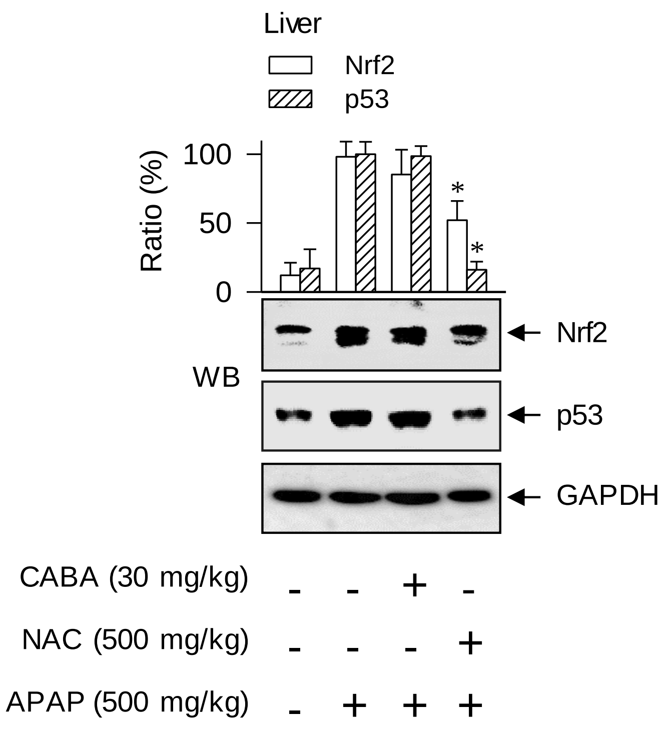

Figure S5. C57BL6 mice were treated with CABA at 1 h after APAP overdose. Liver lobules were biopsied at the time lapse of 15 h. Protein extracts were subjected to Western blot (WB) analysis with anti-Nrf2, anti-p53 or anti-GAPDH antibody. *$P < 0.05$ vs. APAP alone.
